# Hybrid sterility in crosses between two Brazilian sibling species of the *Anopheles albitarsis* complex

**DOI:** 10.1186/s13071-014-0559-6

**Published:** 2014-12-04

**Authors:** Nathalia Giglio Fontoura, Alejandra Saori Araki, Renata Van Der Maas Azevedo, Allan Kardec Ribeiro Galardo, Alexandre Afranio Peixoto, José Bento Pereira Lima

**Affiliations:** Laboratório de Fisiologia e Controle de Artrópodes Vetores, Instituto Oswaldo Cruz, FIOCRUZ, Rio de Janeiro, Brasil; Laboratório de Biologia Molecular de Insetos, Instituto Oswaldo Cruz, FIOCRUZ, Rio de Janeiro, Brasil; Instituto de Pesquisas Científicas e Tecnológicas de Estado do Amapá, Macapá, Amapá Brasil; Laboratório de Entomologia, Instituto de Biologia do Exército, Rio de Janeiro, RJ Brasil

**Keywords:** *Anopheles albitarsis* complex, *Anopheles albitarsis s.s*, *Anopheles marajoara*, Hybrid sterility, Haldane’s rule, Malaria, Speciation

## Abstract

**Background:**

Complexes of cryptic species are common in several taxa and this is also the case in the *Anopheles* genus, a group including all known human malaria vectors. The *Anopheles albitarsis* complex comprises at least nine cryptic species, some of which are implicated as vectors of human malaria. Several different types of data have been generated for this species complex such as cytogenetics, alloenzymes, morphological and feeding behavioral, hybridization experiments, RAPD-PCR and RFLP and mitochondrial and nuclear markers. Studies focused on its postzygotic isolation are still somewhat rare in the literature despite their importance to understand the speciation process and the level of gene flow potentially occurring among the different sibling species.

**Methods:**

Hybridization experiments between *Anopheles albitarsis s.s.* and *Anopheles marajoara*, as well as backcrosses between hybrids and *Anopheles albitarsis s.s.*, were performed using the induced mating technique. Results were compared to intraspecific crosses. Larva-to-adult viability and sex ratio were also assessed.

**Results:**

Male hybrids show very low insemination rates and nearly complete sterility, apparently due to abnormalities in their reproductive organs. Evidence of partial sterility among the hybrid females was also observed.

**Conclusions:**

Our data indicated that *Anopheles albitarsis s.s.* and *Anopheles marajoara* show a high level of postzygotic isolation with a strong hybrid male sterility. This result is consistent with the Haldane’s rule which states that in interspecific crosses the heterogametic sex is the first to be affected. However, the fact that the females are not completely sterile raises the possibility of introgression between these two siblings species.

## Background

Complexes of cryptic species are common in several taxa [1,2] and this is the case in the *Anopheles* genus, a group including all known human malaria vectors [3,4]. A better understanding of cryptic species can be epidemiologically relevant since it could offer clues about differential vector capacity (reviewed in [5]).

*Anopheles albitarsis s.l.* Lynch-Arribálzaga is widely distributed from northern Guatemala to northern Argentina [6] and is a complex of cryptic species that includes some of the most important regional vectors in America [7-9]. The first indication of *An. albitarsis s.l.* occurring as a species complex was described by Kreutzer *et al*. [10]. Since then, several approaches were applied to investigate and characterize the species members: cytogenetics, alloenzymes, morphological and feeding behavioral analysis, hybridization experiments, RAPD-PCR and RFLP and DNA sequencing of mitochondrial and nuclear markers [6,10-20].

Current knowledge suggests that the *An. albitarsis* complex comprises at least nine species: (i) *An. albitarsis s.s.* Lynch-Arribálzaga or *An. albitarsis* A, found in Argentina, Paraguay and Brazil (States of Bahia, Paraná, Santa Catarina, and São Paulo); (ii) *Anopheles oryzalimnetes* Wilkerson & Motoki or *An. albitarsis* B, widespread in Paraguay and probably the species with the wider geographical distribution in Brazil (States of Bahia, Ceará, Espírito Santo, Mato Grosso, Pará, Paraná, Rio de Janeiro and São Paulo); (iii) *Anopheles marajoara* Galvão & Damasceno or *An. albitarsis* C, found in Paraguay, Venezuela and in Brazil (States of Amapá, Amazonas, Mato Grosso, Pará, Rondônia and São Paulo); (iv) *Anopheles deaneorum* Rosa-Freitas or *An. albitarsis* D, distributed in northern Argentina and in Brazil (States of Acre, Mato Grosso, Paraná and Rondônia); (v) *Anopheles janconnae* Wilkerson & Sallum or *An. albitarsis* E, found in northern Brazil (States of Amapá, Pará and Roraima) and in Venezuela; (vi) *An. albitarsis* F, in Colombia, Venezuela and Trinidad; (vii) *An. albitarsis* G, exclusive to Brazil (States of Amazonas, Bahia and Pará); (viii) *An. albitarsis* H, also restricted to Brazil (States of Mato Grosso and Rondônia); and (ix) *An. albitarsis* I, found in Colombia and Venezuela [9,17,20-24]. Moreover, the number of species within the *Anopheles albitarsis* complex can be even higher as *An. deaneorum* and *An. marajoara* might include more than one sibling species [9,25].

In an evolutionary context, analysis of the reproductive isolation between cryptic species of insect vectors allows the identification of potential gene flow among siblings. In addition, laboratory crossing experiments are likely to reveal the exchange of genes potentially involved in vectorial capacity or insecticide resistance, being therefore important for vector control programs [16,26-28]. In the *Albi tarsis* species complex only two hybridization studies between siblings have been published so far [16,29]. This is probably due to the difficulty of keeping these species in captivity, since neotropical *Anopheles albitarsis* species rarely perform free mating crosses under laboratory conditions. In 1991, Klein *et al*. [29] observed male hybrid sterility in crosses between *An. deaneorum* and a species that they called “B”, later defined as *An. marajoara* (Richard C. Wilkerson, personal communication). In the second study, Lima *et al*. [16] observed that hybrid males from crosses between *An. albitarsis s.s.* and *An. deaneorum* showed very low insemination rates and suggested that the abnormalities in their reproductive organs could explain this. In the present work, we performed crossing experiments between two Brazilian sibling species of the *An. albitarsis* complex, *An. albitarsis s.s.* and *An. marajoara*. We used these two species in order to analyze the degree of reproductive isolation between them and compare with previous ones. The present work represents an interesting study from an evolutionary point of view of *An. albitarsis* complex and is epidemiologically relevant focusing a Neotropical malaria vector such as *An. marajoara*.

## Methods

### Mosquito samples

We analyzed specimens of *An. albitarsis s.s.* from a colony maintained at the Instituto de Biologia do Exército (Rio de Janeiro State, Brazil) following the protocol described by Horosko *et al.* [30] with slight modifications. The colony was established in 1993 with mosquitoes collected from Massaranduba municipality (26° 36′ 39″ S, 49° 0′ 33″ W) (Santa Catarina State, Brazil) [16]. This species was treated as a control in crosses since the specimens used were obtained from a stable colony [16]. In addition, *An. marajoara* females were collected in Mazagão municipality (0° 6′ 58″ S, 51° 17′ 10″ W) (Amapá State, Brazil). Specimens were identified as *Anopheles albitarsis s.l.* according to Faran & Linthicum [31] and were considered as *An. marajoara* based on Conn *et al.* [7]. The identification was then confirmed by DNA barcoding (see below). Laboratory rearing of *An. marajoara* was performed according to Lima *et al.* [16]. The *An. marajoara* specimens belonging to F1-F3 generations were used in crossing experiments. All procedures were carried out under controlled conditions: 27°C for the immature stages (larvae to pupae) and 25°C and 70% relative humidity for the adult mosquitoes.

### Identification by DNA barcoding

DNA barcodes (658 bp of the mtDNA Cytochrome c Oxidase – COI) were generated for 11 *An. albitarsis s.s.* and 12 *An. marajoara* specimens (Accession numbers: KM391793 to KM391815). DNA extraction was carried out as in Jowett [32] and PCR amplification using the LCO1490 and HCO2198 primers of Folmer et al. [33]. Sequencing reactions were carried out in both directions with the Big Dye Terminator Kit and ABI 3730 automated sequencer (PE Applied Biosystems). Sequences were edited in Bioedit 7.2.3 [34] and the delineation of species within Albitarsis group by phylogenetic analysis was carried out using specimens from each distinctive COI lineage found by NJ-K2P analysis. The sequences added were: *An. albitarsis* H: GenBank: DQ076222, DQ076223, DQ076224; *An. deaneorum*: GenBank: DQ076226, DQ076227, DQ076229, DQ076230; *An. albitarsis* G: GenBank: DQ076221, DQ076225; *An. oryzalimnetes*: GenBank: DQ076210, DQ076211, DQ076213, DQ0762105. We used the Kimura-two-parameter distance model (K2P), Neighbor-joining analysis (NJ) and 1,000 replicates to produce an unrooted consensus tree.

### Crossing experiments

Methods for crossing experiments were performed as described in Lima *et al.* [16]. Males and females were separated every twelve hours to guarantee the collection of virgin females [35]. Females were fed according to the protocol approved by the Fiocruz Ethical Committee on Use of Animals (CEUA) and subjected to the artificial-mating technique [36]. Four days after mating, the females were placed in a plastic device (8.5 cm-diameter × 4.5 cm-depth) closed with a nylon mesh and submerged in a bowl (18 cm-diameter × 7.5 cm-depth) filled with dechlorinated water. The internal surface of the plastic device was covered with moist filter paper to keep eggs from drying out. After two days, the spermathecae (from alive and dead females) were dissected and examined by light microscopy; mating success was scored based on the presence of sperm.

Eggs were counted after forced egg laying. Two days after hatching, larvae were counted and transferred to water-filled plastic bowls. Every two days numbers of larvae and living pupae were counted. The immature stages were reared following the same protocol already mentioned for the colony maintenance. Adults were used in the subsequent crosses. Intra and interspecific mating experiments were performed and the hybrid offspring were used in backcrosses according to Lima *et al*. [16]. The parameters evaluated for each crossing experiment were: the number of inseminated females, the total number of eggs, hatching eggs, larvae, pupae and adults, and also the male and female ratio.

### Evaluation of the male genital apparatus

Male genital structures of the offspring were dissected and examined using stereoscopy microscopy. External and internal genital structures were mounted in slides with saline solution, and in some cases, were stained using the buffer reference standard pH = 4.0 (Sigma) for a better visualization. Chi-square statistical analysis was estimated by the program GraphPad Prism 5.0 [37].

## Results

### DNA barcoding

**Figure 1:**
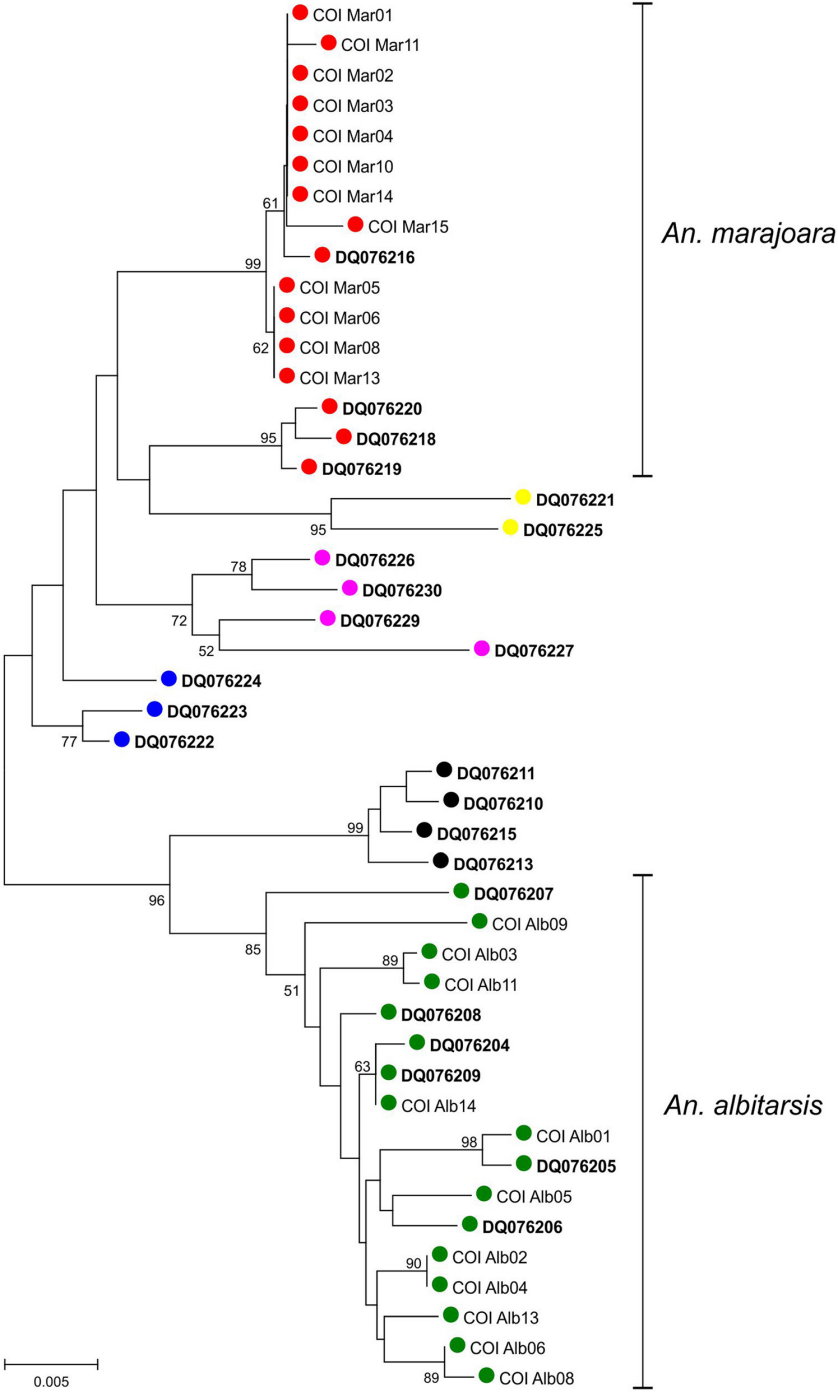
**Phylogenetic tree generated using GeneBank sequences and specimens of** ***An. marajoara*** **and** ***An. albitarsis s.s.*** The lineages are represented in different colors: 1) *An. albitarsis* H in blue, 2) *An. deaneorum* in pink; *3*) *An. albitarsis* G in yellow; 4) *An. oryzalimnetes* in black; 5) *An. albitarisis s.s*. in green and 6) *An. marajoara* in red. Bootstrap values were obtained with 1,000 permutations. Only values above 50% are shown.

Figure 1 shows a phylogenetic tree with six clusters clearly defined: 1) *An. albitarsis* H; 2) *An. deaneorum*; 3) *An. albitarsis* G; 4) *An. oryzalimnetes*; 5) *An. albitarisis s.s*. and 6) *An. marajoara*. The latter group included all individual sequences of *An. marajoara* showing that we used a pure lineage on our crossing experiments.

### Viability assessment

Table 1 shows larvae-to-adult viability of the offspring from intraspecific and interspecific crosses between *An. albitarsis s.s.* and *An. marajoara*, and also backcrosses of these hybrids with *An. albitarsis s.s*.. In each case, eight different crosses were conducted. The hatching number from the different groups of crosses was highly variable. The two intraspecific and the interspecific crosses between male *An. marajoara* × female *An albitarsis s.s.* showed very similar hatching percentages (ranged between 38% and 45%). Nevertheless, a remarkable reduction on hatching rates was observed in all backcrosses. Furthermore, crosses between hybrid males and *An. albitarsis s.s.* females resulted in null or near to zero rates. In general, the pupation was a more homogeneous developmental event than hatching. A significant reduction of the larvae viability was observed in crosses that involved females of *An. marajoara,* in conspecific crosses of *An. marajoara* (*X*^2^ = 26.34, df = 1, *P* < 0.001) and male *An. albitarsis s.s.* × female *An. marajoara* (*X*^2^ = 4.48, df = 1, *P* = 0.034). Differing from the dramatically decreased hatching in backcrosses, pupation and adult emergence seemed unaffected when hybrid females were used. Intraspecific and interspecific crosses also resulted in similar and high percentages of emergence. Finally, we observed that in all cases the sexual ratio was not statistically different from 1:1 indicating similar mortality between genders.

**Table 1:** **Larvae-to-adult viability of the intraspecific offprings and hybrids between species of the** ***Anopheles albitarsis*** **complex**

**Crosses**	**Total of eggs N**	**Hatching N (%)**	**Pupation N (%)**	**Adult emergence N (%)**	**Alive (%)**	**Males N (%)**	**Females N (%)**	**M/F ratio**
Intraspecific								
♂ALB × ♀ALB	2128	887 (41.7)	475 (53.6)	392 (82.5)	(44.2)	192 (49.0)	200 (51.0)	0.96
♂MAR × ♀MAR	2733	1048 (38.3)	258 (24.6)***	204 (79.1)	(19.5)	85 (41.7)	119 (58.3)	0.71
Interspecific								
♂ALB × ♀ MAR	3812	2194 (57.6)***	861 (39.2)*	740 (85.9)	(33.7)	404 (54.6)	336 (45.4)	1.20
♂MAR × ♀ ALB	2352	1057 (44.9)	555 (52.5)	406 (73.2)	(38.4)	197 (48.5)	209 (51.5)	0.94
Backcrosses								
♂ALB × ♀Hybrid A	1479	143 (9.7)***	76 (53.1)	57 (75.0)	(39.9)	29 (50.9)	28 (49.1)	1.04
♂ALB × ♀Hybrid B	2489	210 (8.4)***	111 (52.9)	78 (70.3)	(37.1)	39 (50.0)	39 (50.0)	1.00
♂Hybrid A × ♀ALB	1823	0	0	0	0	0	0	0
♂Hybrid B × ♀ALB	921	1 (0.001)	0	0	0	0	0	0

N, number; (%), percentage in brackets; ALB, *Anopheles albitarsis s.s.*; MAR, *Anopheles marajoara*; Hybrid A: resulting of interspecific crosses between ♂ALB × ♀MAR; Hybrid B: resulting of interspecific crosses between ♂MAR × ♀ALB.

*P < 0.05; ***P < 0.001.

### Evaluation of insemination

After oviposition, the eggs were counted and the spermathecae dissected and classified as positive or negative depending on the presence or lack of sperm, respectively. Females with positive spermathecae were considered inseminated. Table 2 shows the number of mated and positive females for each performed cross. As expected, a higher percentage of inseminated females was observed in crosses involving *An. albitarsis s.s*. males since they result from induced mating, which introduces a level of selection [38]. The low *An. marajoara* male effectiveness can be attributed to the use of F1 and F2 mosquito generations not adapted to laboratory conditions. The crosses with *An. marajoara* males were really challenging, even compared with other Anopheline species, such as *An. deaneorum.* The number of inseminated females was significantly lower in crosses with hybrid males than with *An. albitarsis s.s.* In some cases, females mated with interspecific males showed immotile sperm or even agglomerated sperm with dark pigmentation (data not shown).

**Table 2:** **Insemination rates in crosses between species of the** ***Anopheles albitarsis*** **complex and their reciprocal hybrids**

**Crosses**	**Mated females N**	**Spermathecae** ^**+**^ **N (%)**
Intraespecific		
♂ALB × ♀ALB	125	65 (52.0)
♂MAR × ♀MAR	166	35 (21.1)**
Interspecific		
♂ALB × ♀ MAR	168	76 (45.2)
♂MAR × ♀ALB	195	35 (17.9)**
Backcrosses		
♂ALB × ♀Hybrid A	95	49 (51.6)
♂ALB × ♀Hybrid B	111	55 (49.5)
♂Hybrid A × ♀ALB	133	2 (1.5)***
♂Hybrid B × ♀ALB	98	6 (6.1)***

N, number; (%), percentage in brackets; ^+^positive spermathecae (with sperm in its interior); ALB, *Anopheles albitarsis s.s*.; MAR, *Anopheles marajoara*; Hybrid A: resulting of interspecific crosses between ♂ALB × ♀MAR; Hybrid B: resulting of interspecific crosses between ♂MAR × ♀ALB. **P < 0.01; ***P < 0.001.

**Figure 2:**
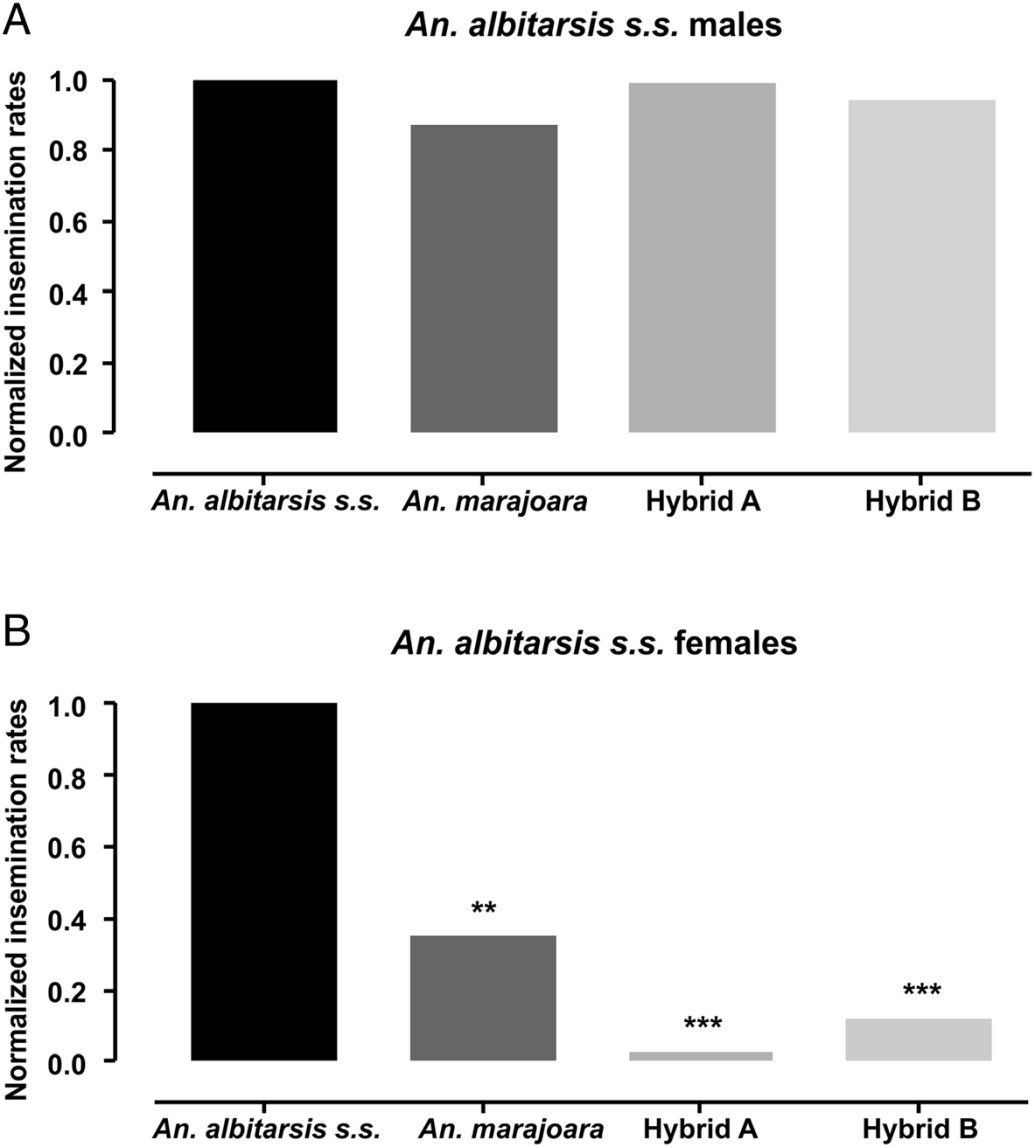
**Normalized insemination rates. (A)** Crosses between females of different genotypes and *Anopheles albitarsis s.s.* males and **(B)** between males of different genotypes and *Anopheles albitarsis s.s.* females. **P < 0.01; ***P < 0.001.

Figure 2 shows comparisons between different crosses using the normalized insemination rates estimated from Table 2 data. Normalization was carried out using results from the intraspecific cross of *An. albitarsis s.s.*. Figure 2A illustrates the normalized insemination rates involving females of different genotypes crossed to *An. albitarsis s.s.* males. In all cases, the difference between female insemination rates was not significant, regardless of their genotype. The results observed for the two types of hybrid females were expected since they have a similar genotype. Figure 2B shows the normalized insemination data of *An. albitarsis s.s.* females mated to males of different genotypes. Comparing to the intraspecific cross, a significant reduction of insemination rates was observed in all other crosses. *Anopheles marajoara* males inefficiently inseminate *An. albitarsis s.s*. females (*P* < 0.01) and hybrid males performed even less successfully (*P* < 0.001). However, insemination rates of crosses using both types of hybrid males did not differ significantly. Although some females inseminated by hybrid males laid eggs, their viability was null (see Table 1).

**Figure 3:**
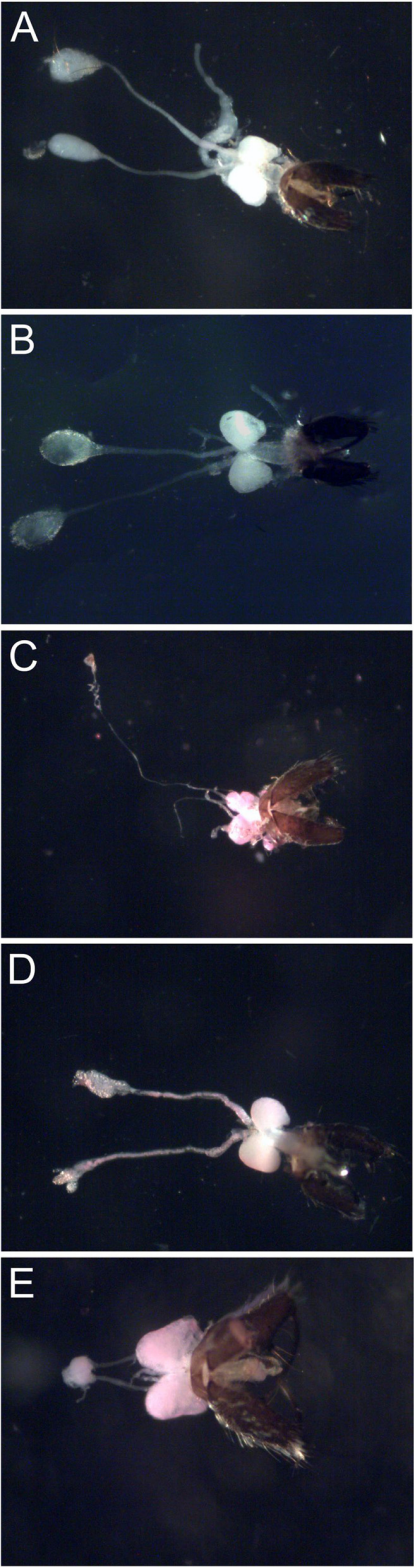
**Male reproductive organs showed malformations on testis and vas deferens. (A)** *Anopheles albitarsis s.s.*, **(B)** *Anopheles marajoara* and **(C-E)** hybrids.

Figure 3 shows the male reproductive organs of *An. albitarsis s.s.* and *An. marajoara* (Figure 3A and B, respectively) and hybrids resulted from interspecific crosses (Figure 3C-E). Invariably, abnormalities were detected only in male hybrids. Malformations consisted of reduced testis with long and thin vas deferens (Figure 3C), abnormal testis with a lobular structure and a malformed and irregular vas deferens (Figure 3D) or fused testis with short vas deferens (Figure 3E).

## Discussion

Variable patterns of reproductive isolation are observed between pairs of siblings of Anopheles species complexes. For example in the *An. gambiae* complex, two molecularly distinguished forms, known as M and S, do not show reduction in hybrid males and females’ viability or fertility. This result suggests that other mechanisms, apart from postzygotic developmental barriers [39], are acting in this speciation process. In this sense, it has been claimed that differences in wing-beat frequencies recognition plays a relevant role [40]. Hybridization between *Anopheles minimus* A and C, from the *An. minimus* complex, results in fertile males and females with normal ovaries [41]. However, crosses between *An. minimus* A and E produce sterile males and normal females [42].

Concerning the postzygotic isolation mechanisms, the hybrid sterility seems to be one of the first outcomes observed in anophelines [16,29,42-46]. Crosses between *An. albitarsis s.s*. and *An. marajoara* produced viable hybrid offspring from both genders in normal rates suggesting the absence of prezygotic isolation mechanisms. However, our results point to a high degree of postzygotic reproductive isolation between these sibling species. The observed pattern of crosses fits the Haldane’s rule, which states that in interspecific crosses the heterogametic sex shows sterility or viability problems before the homogametic one [47]. Eggs resulted from crosses involving hybrid males do not hatch at all. This indicates that the reproductive isolation is a consequence of males’ sterility, possibly due to abnormalities in their reproductive organs. Although hybrid females exhibit a similar degree of insemination when compared to *An. albitarsis s.s.* control, the hatching percentage of their eggs also suggests postzygotic isolation. Moreover, this isolation phenomenon seems to have a lower effect in females than in males. Interestingly, the development rate of the hybrid females’ offspring is comparable to the intraspecific *An. albitarsis s.s*. control. This indicates some degree of hybrid females’ infertility, a potential further point of control of interspecific gene flow, at the embryonic stage. In the *An. albitarsis* complex, previous analysis of reproductive isolation between *An. albitarsis s.s*. and *An. deaneorum*, also revealed postzygotic isolation due to sterility of hybrid males [16]. Differing from the present observations, those hybrid males exhibited different levels of sterility. In that case, hybrid males carrying an *An. deaneorum* X chromosome were the most affected. Moreover, *An. deaneorum* males were less successful in inseminating *An. albitarsis s.s* females than their conspecific females, whereas no significant difference was observed in the reciprocal cross. This result suggests that in areas where both species occur in sympatry, asymmetrical introgression might happen. This asymmetry in the hybrid sterility is frequent in interspecific crosses of closely related species [48]. In spite of that, our results suggest an established and effective barrier to this interspecific introgression considering the almost complete failure of both *An. albitaris s.s*. and *An. marajoara* males. Further crossing experiments and studies of natural populations using molecular markers might help to determine the genetic relationships and the gene flow among siblings of the Albitarsis complex that includes some of the malaria vectors of America.

## Conclusions

Our data confirm that *An. albitarsis s.s*. and *An. marajoara* are distinct species of the Albitarsis complex with a high degree of postzygotic reproductive isolation between them. Hybrid males show sterility probably caused by abnormalities in their reproductive organs and a subtle effect was observed in the hybrid females. This result is consistent with the Haldane’s rule which states that in interspecific crosses the heterogametic sex is the first to be affected. The fact that the females are not completely sterile raises the possibility of introgression between these two siblings species.
